# Enhancing semantic congruity effects with category-contingent comparative judgments

**DOI:** 10.3389/fpsyg.2014.01199

**Published:** 2014-10-22

**Authors:** Craig Leth-Steensen, William M. Petrusic, Samuel Shaki

**Affiliations:** ^1^Department of Psychology, Carleton UniversityOttawa, ON, Canada; ^2^Department of Behavioral Sciences, Ariel University Center of SamariaAriel, Israel

**Keywords:** symbolic comparison, perceptual comparison, semantic congruity effect, evidence accrual, comparative instruction manipulation

## Abstract

In each of two experiments the direction of a binary comparison was contingent on the category of the stimulus pair. In one experiment, participants had to compare the size of animals from memory. On congruent trials, they had to select the smaller animal if both were small and the larger if both were large and on incongruent trials they selected the larger if both were small and the smaller if both were large. In a second experiment, participants had to compare visual extents and the direction of the comparison was contingent on whether the lines were short or long. Response times were increased and semantic congruity effects (SCEs) were greatly amplified with the category-contingent instructions relative to the conventional non-contingent instructions, precisely as predicted by the class of evidence accrual models of decisional processing and contrary to the single-sample stage models of the SCE.

## INTRODUCTION

Semantic congruity effects (SCEs) reflect a robust and enduring property of comparative judgments involving both perceptual and symbolic stimuli. As in the present experiments, SCEs are evident when the selection of the smaller of two relatively small stimuli is faster than the selection of the larger and, conversely, when selection of the larger of two relatively large stimuli is faster than selection of the smaller. The SCE poses an ongoing challenge to all currently available theories of the comparison process. A key aspect of this effect is its inherent illogicality. Namely, regarding something as being the smaller of two things is logically equivalent to regarding the other as being the larger, hence, no information processing differences between these two types of comparison conditions would be expected (i.e., they should be interchangeable but are not). Although a number of theories have specifically been proposed as accounts for this effect in the past, incorporating them into the currently available, more formal, comparison models (e.g., Usher and McClelland, 2001; Ratcliff and Smith, 2004: Verguts et al., 2005; Brown and Heathcote, 2008) is not trivial and, for the most part, has not been attempted (except by the current authors).

In the typical psychophysical comparison experiment, the instructions specifying the direction of the comparison are explicit and defined by the instruction presented. In the present experiments, we made the direction of the comparison dependent on the outcome of a binary categorization. On half of the trials, the direction was semantically congruent with the category and on the other half it was semantically incongruent. In particular, in one experiment, using pairs of line lengths varying from short to long, we required participants to select the shorter line if both lines were short and the longer line if both were long on congruent trials. On incongruent trials, participants had to select the longer line in the pair if both lines were short and the shorter line if both lines in the pair were long. We reasoned that the magnitude of the SCE when the direction of the comparison was contingent on the category of the stimulus pair relative to its magnitude when the usual instructions were presented would be theoretically revealing about both the basis for the SCE and, more generally, the processes involved in comparative judgments.

Petrusic (1992), in the context of his Slow and Fast Guessing Theory, presented experimental evidence in support of the notion that the SCE occurs at the level of each pass through the evidence accumulation process. Notably, he showed that the properties of the SCE mirror the properties of the underlying response times (RTs). For example, under conditions emphasizing accuracy at the expense of speed, RTs on error trials are longer than on correct trials, and concomitantly, the SCE is larger on error trials than on correct trials. Conversely, under speed stress RTs on error trials are as fast or faster than on correct trials and again the size of the SCE mirrors this pattern. Moreover, in an experiment with varying deadlines for speed versus accuracy, SCEs increased approximately linearly with the base time arising from the deadlines but more so for error trials than for correct trials. On the basis of these findings, Petrusic (1992) argued that the duration of each accrual is longer when the stimulus pair location and the direction of the comparison are incongruent.

Findings from two studies involving two rather different instructional manipulations have converged to further implicate an evidence accrual basis for the SCE. First, Shaki et al. (2006) found that the SCE was larger when instructions varied randomly from trial to trial as compared to when they were fixed for an entire (large) block of trials. Next, Petrusic et al. (2008) showed that SCEs were reliably larger when the instructions indicating the direction of the comparison were represented by consonant-vowel-consonant (CVC) nonsense syllables (that had been associated with the usual instructions in a preliminary learning phase of the experiment) than with the usual instructions.

On Petrusic’s (1992) view that the SCE occurs at the level of each pass through the evidence accumulation process, enhanced SCEs are predicted when either the instructions are randomized from trial to trial or when they are presented as CVCs given that in both cases, the activation of the underlying semantic instruction is degraded resulting in further slowing of the evidence accrual process. With respect to the category-contingent instructional manipulations in this current study, according to Petrusic’s (1992) view, if it is the case that the comparative instruction must be reactivated on each evidence accrual event and that such reactivation sometimes also involves having to reinstate the stimulus-based categorical information in the category-contingent instructional condition, such a state of affairs would then be expected to drastically slow down the evidence accrual process and, hence, greatly enhance the SCE in that condition.

Similarly, both Shaki’s et al. (2006) and Petrusic et al’s. (2008) results can also be accounted for by a related evidence accrual model, the Leth-Steensen and Marley (2000) connectionist, instructional pathway, interference model. Namely, by assuming that either randomizing the instructions or presenting them as CVCs leads to an increase in instructional pathway competition. Within Leth-Steensen and Marley (2000) model, responses are based on activation accumulating simultaneously within two competing instructional pathways, one associated with the relevant instruction and one associated with the irrelevant instruction. The SCE is assumed to arise from the fact that the strength of activation within each of these pathways is modulated by the relative size of the stimulus pair such that the strength of the congruent instructional pathway is enhanced. With respect to the category-contingent instructional manipulations in this current study, according the Leth-Steensen and Marley (2000) model, making the direction of the comparison dependent on the result of a binary categorization should then serve to enhance the processing requirements associated with maintaining that instruction in memory as a contextual guide to the comparison process. Such a state of affairs would then be expected to increase the level of instructional pathway competition within the model resulting in a larger SCE.

Hence, demonstrating that the use of category-contingent instructions enhances the size of the SCE would provide further support for an evidence accrual view of the SCE as exemplified by either the Petrusic (1992) view or the Leth-Steensen and Marley (2000) model. As will be discussed, such a result cannot be accounted for by any of the available single-sample (i.e., non-evidence-accrual-based) accounts for this effect. In the following, category-contingent directional comparisons are used, first, for comparisons of symbolic stimuli (i.e., animal sizes) and, second, for comparisons of perceptual stimuli (i.e., line lengths). In addition to providing an explicit replication of the symbolic stimulus comparison results (for which SCEs have most commonly been demonstrated in the literature), the use of line lengths as perceptual comparison stimuli allows for a more rigorous control over the discriminative difficulty of the comparisons.

## EXPERIMENT 1

### METHOD

#### Participants

Nineteen Carleton University students participated in one 40-min session to satisfy course requirements and all reported normal or corrected-to-normal vision. The running of this experiment (and the following one) was approved by the Carleton University Psychology Research Ethics Board and treatment of all participants conformed to the standards set out by the American Psychology Association.

#### Apparatus

Graphics production, presentation of instructions and stimuli, event sequencing and timing, and the recording of responses and RTs were controlled by Pentium III computer running under SuperLab control. Stimuli and instructions were presented on a 17 inch (43 cm) ViewSonic video monitor with 800 by 600 pixel resolution. Responses were made using the buttons on an IBM-PC Mouse with the roller-ball disabled.

#### Stimuli and design

Six animal names, all three-letter words in English, printed in Times New Roman font (25, bold) defined the stimulus set. Three names were of relatively small animals (ant, bee, bat), whereas the other three names were of relatively large animals (dog, pig, cow). The animal names were paired within categories resulting in three relatively small animal pairs (ant-bee, ant-bat, bee-bat), and three relatively large animal pairs (dog-pig, dog-cow, pig-cow). On each trial, a pair was presented at the respective centers of the left and right hemi-fields of the computer screen on a white background. Each pair in the design was presented in each of the two possible left-right position orders, resulting in 12 animal names pairs.

The two words “Larger” and “Smaller” (in the usual condition), and the two sentences “small-smaller but large-larger,” and “small-larger but large-smaller” (in the category-contingent condition) served as the comparative instructions. These instructions were printed in Times New Roman font (size 30, bold) and were always displayed at the center of the upper third of the screen. Both the two instructional conditions and the two forms of comparative instructions within each condition occurred equally often and appeared randomly from trial to trial. This factorial combination (the 12 stimulus pairs by two instruction direction by two instructional conditions) was replicated six times, preceded by one replication of practice trials. The participants were not aware of the partition into practice and experimental trials. The order of presentation of the stimulus pairs within blocks was random and different for each participant.

#### Procedure

Participants were tested individually in a dimly lit room, seated ∼80 cm from the center of the video monitor. In the usual instruction condition, participants were told that the presentation of either the word “Smaller” or “Larger” served as a warning for the next trial and was an instruction that indicated whether they were to choose either the larger or the smaller animal in the upcoming pair. As well, they were told that the appearance of the sentence “small-smaller, but large-larger” means if both animals in the pair are relatively small they are to pick the smaller animal, but if both animals are relatively large they are to pick the larger animal. Similarly, they were told that the instruction “small-larger, but large-smaller” served as an instruction to choose the larger animal in the pair if both animals are relatively small, or the smaller animal if both animals are relatively large. After an additional 750 ms, the pair of animal names appeared while the comparative instruction remained on the screen. The participant’s task was to press the left or the right button of a mouse corresponding to the side of the larger (or smaller, respectively) member of the pair of animal names, according to the appropriate instruction.

The presentation of the stimuli and the comparative instruction was response-terminated and the next trial began 1000 ms later. Participants were encouraged to respond quickly, but accurately. The session included three planned breaks, which ended with the participants’ decision to continue.

### RESULTS

An analysis of variance (ANOVA) was performed with block (six levels), stimulus pair (six pairs), instruction direction (two levels), and instructional condition (two levels) as factors. The dependent variable was the mean RT for each participant in each cell of the design. The Huynh-Feldt epsilon adjustment to the degrees of freedom was used to test each effect. Level of significance was set at 0.05 throughout. As expected with symbolic comparisons of this nature, error rates were quite low. In the usual instructions condition the error rate was 2.96% whereas in the category-contingent instructions condition it was 4.04%.

**FIGURE 1:**
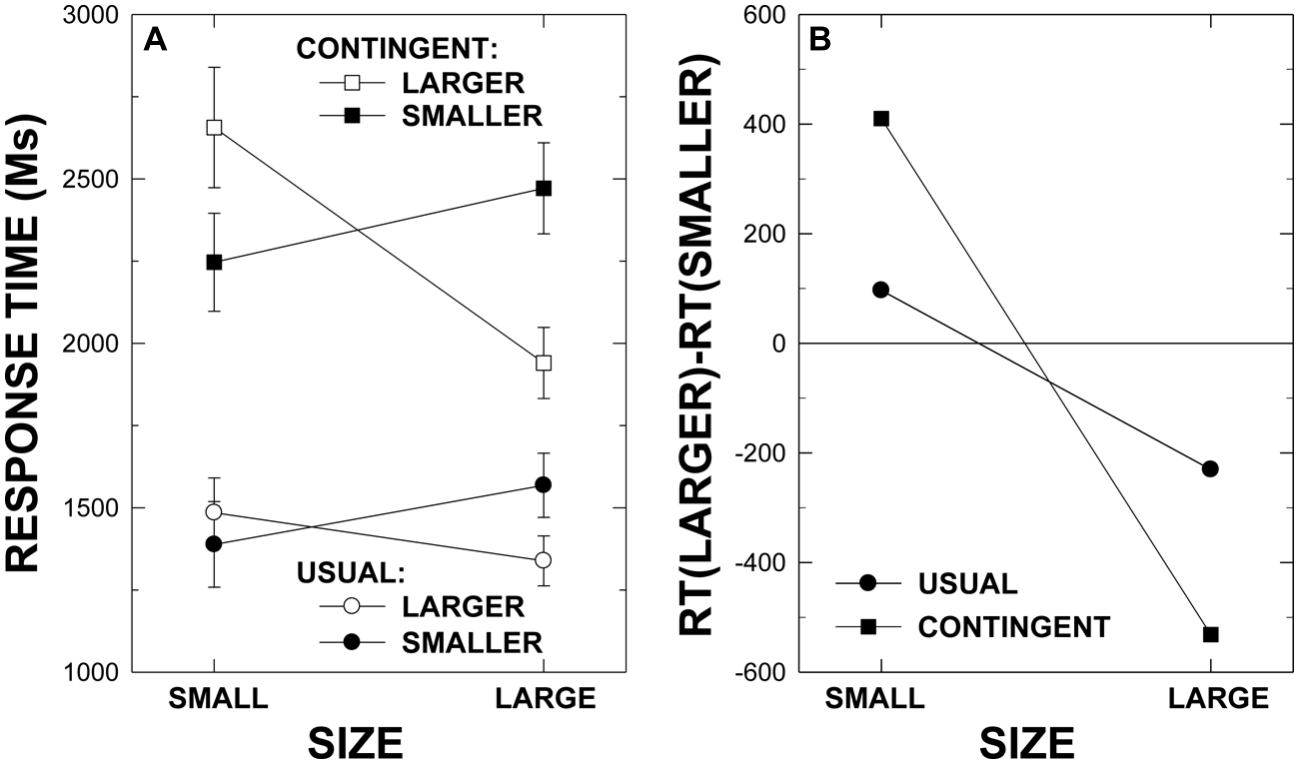
**Mean response times and standard error bars for the small and large animal size stimulus pairs with each instruction in the usual and category-contingent instruction conditions **(A)** in Experiment 1.** The semantic congruity index, defined by RT (“Larger”) – RT (“Smaller”), for the small and large animal size pairs with the usual and the category-contingent instructions is provided in **(B)**.

**Figure 1A** provides a view of the main features of the results of this experiment, providing means and error bars for the sets of small and large pairs. As is evident from these plots, the category-contingent instructional manipulation proved extremely effective in slowing the comparison process. Mean RTs are 1448 ms (SE = 99) in the usual instructions condition and 2330 ms (SE = 138) in the category-contingent instructions condition. An ANOVA showed this difference to be reliable, *F*(1,18) = 204.11, *p* < 0.001, $\eta_{p}^{2}$ = 0.919, MSE = 2605115. As well, mean RTs are 1855 ms (SE = 108) with the instruction to select the name of the larger animal and 1922 ms (SE = 125) with the instruction to select the smaller (i.e., a lexical markedness effect; Leth-Steensen and Marley, 2000). This effect of instruction direction is statistically reliable, *F*(1,18) = 6.11, *p* < 0.024, $\eta_{p}^{2}$ = 0.1254, MSE = 501195. The main effect of stimulus pair is also reliable, *F*(3.333, 60.001) = 18.35, *p* < 0.001, $\eta_{p}^{2}$ = 0.505, MSE = 1434209, with mean RTs of 1931 (SE = 158), 1819 (SE = 128), 2083 (SE = 140), 2128 (SE = 120), 1626 (SE = 85), and 1742 ms (SE = 93) for the ant-bee, ant-bat, bee-bat, dog-pig, dog-cow, and pig-cow pairs, respectively. Moreover, stimulus pair effects differed in the category-contingent instructions condition in comparison to the usual instructions condition, *F*(4.825, 86.856) = 7.83, *p* < 0.001, $\eta_{p}^{2}$ = 0.303, MSE = 449260. Namely, mean RTs are 1497 (SE = 148), 1270 (SE = 94), 1555 (SE = 120), 1718 (SE = 104), 1311 (SE = 85), and 1335 ms (SE = 84) in the usual instructions condition and 2366 (SE = 174), 2369 (SE = 170), 2611 (SE = 167), 2538 (SE = 153), 1941 (SE = 95), and 2150 ms (SE = 118) in the category-contingent instructions condition for the ant-bee, ant-bat, bee-bat, dog-pig, dog-cow, and pig-cow pairs, respectively.

Most importantly, as is clearly evident in the plots in **Figure 1A**, the classic SCE crossover pattern is evident in both instructional conditions, and the interaction between stimulus pair and instruction direction, which essentially defines the SCE, is highly reliable, *F*(3.763, 67.733) = 26.78, *p* < 0.001, $\eta_{p}^{2}$ = 0.598, MSE = 786242 [where note that analyses of each instructional condition separately indicated that the pair by instruction effect is reliable for both the usual instructions, *F*(4.586, 82.542) = 11.98, *p* < 0.001, $\eta_{p}^{2}$ = 0.400, MSE = 275321, and the category-contingent instructions, *F*(2.552, 45.932) = 18.08, *p* < 0.001, $\eta_{p}^{2}$ = 0.501, MSE = 1772868, respectively]. It is also clear from these plots that the SCE is greatly enhanced in the category-contingent instructions condition consistent with the evidence accrual views. Indeed, the three-way interaction involving stimulus pair, instruction direction, and instructional condition is statistically reliable, *F*(2.391, 43.040) = 6.25, *p* < 0.003, $\eta_{p}^{2}$ = 0.258, MSE = 1182641.

The plots in **Figure 1B** provide an alternative way of viewing both the crossover SCEs in each condition and the enhanced SCE in the category-contingent instructions condition. These plots are based on the SCE index, defined by RTs with the instruction smaller subtracted from RTs with the instruction larger. The full crossover effect is evident when the SCE is positive (i.e., RTs are longer with the instruction larger than with the instruction smaller) for the relatively small pairs of animals and negative for the relatively large pairs of animals. The enhanced SCE for the category-contingent comparisons is clearly reflected in the uniformly larger (absolute) SCE index values in this condition.

Finally, mean RT reliably decreases across blocks, *F*(3.221, 57.985) = 16.95, *p* < 0.001, $\eta_{p}^{2}$ = 0.485, MSE = 1637474, with mean RTs of 2213 (SE = 158), 2048 (SE = 131), 1794 (SE = 108), 1823 (SE = 112), 1717 (SE = 114), and 1737 ms (SE = 117) for Blocks 1–6, respectively. However, neither the two-way interaction of stimulus pairs with instruction direction nor the three-way interaction involving stimulus pair, instruction direction, and instructional condition interacts reliably with blocks [*F*(13.066, 235.186) = 0.62, *p* < 0.835, $\eta_{p}^{2}$ = 0.046, MSE = 778060, respectively]. Hence, neither the overall size of the SCE nor the difference in the size of this effect across instructional conditions decreases as mean RT decreases across blocks. On the other hand, the overall difference in mean RT between the category-contingent and usual instructions conditions does decrease reliably across blocks, *F*(3.745, 67.413) = 7.20, *p* < 0.001, $\eta_{p}^{2}$ = 0.286, MSE = 497535, with differences of 1111, 1013, 835, 868, 748, and 716 ms for Blocks 1–6, respectively.

## EXPERIMENT 2

The enhanced SCE with the category-contingent instructions provides considerable support for the evidence-accrual-based theoretical accounts of the SCE developed in Leth-Steensen and Marley (2000) and in Petrusic (1992). With a view toward replicating and extending the generality of the findings from Experiment 1, comparisons with perceptual stimuli were required in Experiment 2.

### METHOD

#### Participants

Eighteen Carleton University students participated in one 50-min session to satisfy course requirements. All subjects reported normal or corrected-to-normal vision.

#### Apparatus

The apparatus of Experiment 1 was used in this experiment.

#### Stimuli and design

Twelve horizontal lines were used as the stimulus set. Six lines were relatively short (10, 11, 20, 21, 50, and 51 pixels) and the other six were relatively long (147, 150, 200, 210, 250, and 252 pixels). Three pairs of relatively short lines (10–11, 20–21, 50–51), and three pairs of relatively long lines (147–150, 200–210, 250–252) were created. It is well-known that the difficulty of comparative judgments can be effectively manipulated by varying the ratio of the longer to the shorter extent of the comparison pair (e.g., Münsterberg, 1894; Petrusic and Jamieson, 1979). Hence, the three short stimulus pairs are defined, in terms of difficulty, by the ratios 1.1, 1.05, 1.02, respectively whereas the ratios are 1.02, 1.05, and 1.008 for the three long pairs, respectively. All lines, drawn by Paintbrush software, were 1 mm wide and appeared in black on a white background. The pairs of lines appeared at the respective centers of the left and right hemi-fields on the monitor.

The two words “Longer” and “Shorter” (in the usual instructions condition), and the two sentences “short-shorter, but long-longer,” and “short-longer, but long-shorter” (in the category-contingent instructions condition) served as the comparative instructions. The instructions were printed in Times New Roman font (size 30, bold), and were displayed at the center of the upper-third of the screen. Both the two instructional conditions and the two forms of comparative instructions within each condition occurred equally often and appeared randomly from trial to trial. This factorial combination (the 12 stimulus pairs by two instructions by two instructional conditions) was replicated six times, preceded by one replication of practice trials. The participants were not aware of the partition into practice and experimental trials. The order of presentation of the stimulus pairs within blocks was random and different for each participant.

#### Procedure

Participants were tested individually in a dimly lit room, seated ∼80 cm from the center of the video monitor. In the usual instructions condition, participants were told that the presentation of either the word “Shorter” or “Longer” served as a warning for the next trial and was an instruction that indicated whether they were to choose either the longer or the shorter line in the upcoming pair.

As well, they were told that the appearance of the sentence “short-shorter, but long-longer” means if both lines in the pair are relatively short they are to pick the shorter line, but if both lines are relatively long they are to pick the longer line. Similarly, they were told that the instruction “short-longer, but long-shorter” served as an instruction to choose the longer line in the pair if both lines are relatively short, or the shorter line if both lines are relatively long. After an additional 750 ms, the pair of lines appeared while the comparative instruction remained on the screen. The participant’s task was to press the left or the right button of a mouse corresponding to the side of the longer (or shorter, respectively) member of the pair of lines length, according to the appropriate instruction.

The presentation of the stimuli and the comparative instruction was response-terminated and the next trial began 1000 ms later. Participants were encouraged to respond quickly, but accurately. The session included three planned breaks, which ended with the participants’ decision to continue.

### RESULTS

An ANOVA was performed with block (six levels), stimulus pair (six pairs), instruction direction (two levels), and instructional condition (two levels) as factors. The dependent variable was the mean RT for each participant in each cell of the design. The Huynh-Feldt epsilon adjustment to the degrees of freedom was used to test each effect. Level of significance was set at 0.05 throughout. As expected with these relatively difficult perceptual comparisons, error rates were high. In the usual instructions condition, errors occurred on 30.94% of the trials and on 34.11% of the trials in the category-contingent instructions condition.

**FIGURE 2:**
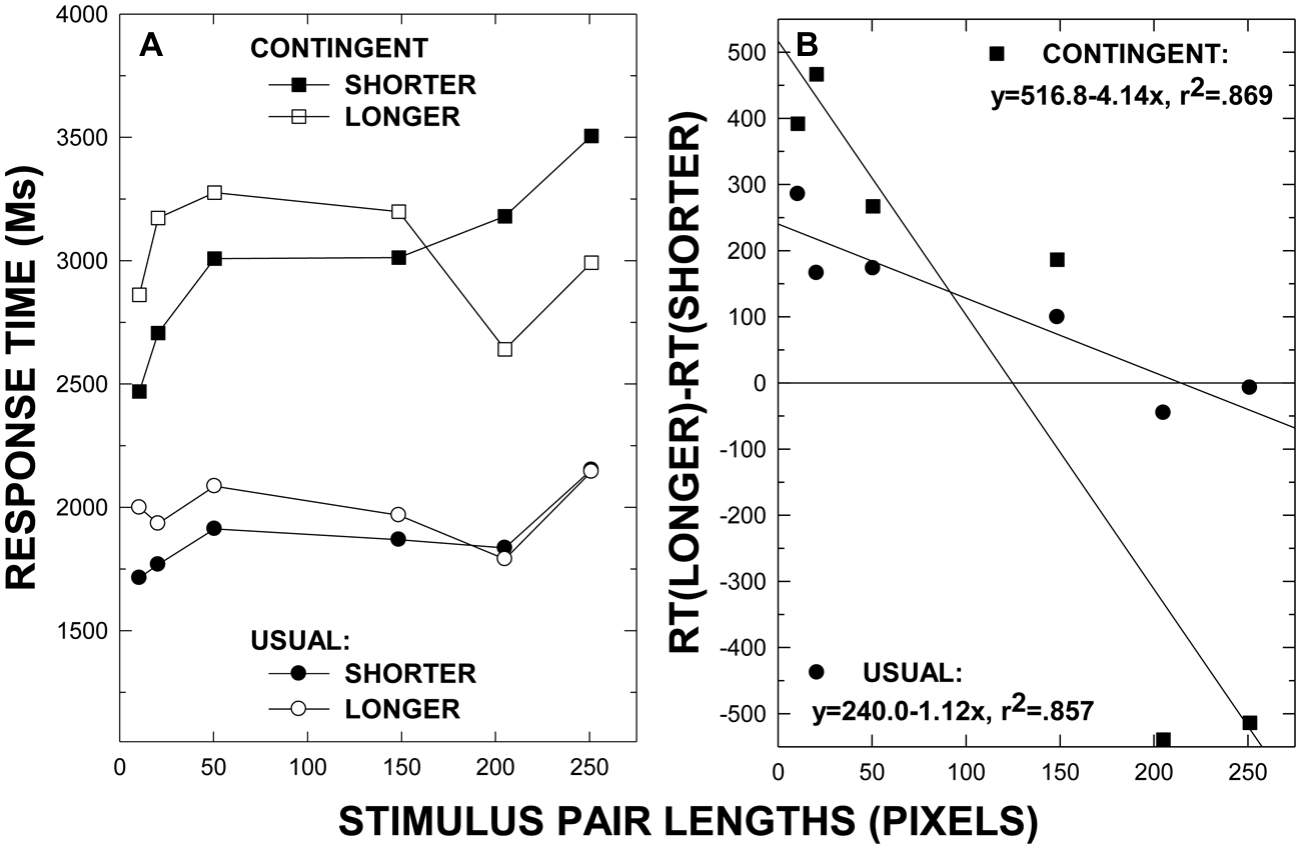
**Mean response times and standard error bars for each visual extent pair in Experiment 2 with each instruction in the usual and category-contingent instruction conditions **(A)**.** The semantic congruity index, defined by RT (“Longer”) – RT (“Shorter”), for the each stimulus pair with the usual and the category-contingent instructions is provided in **(B)**.

As is clear from the plots in **Figure 2A**, and as in Experiment 1, the category-contingent comparisons are considerably and reliably longer (3065 ms; SE = 243) than the comparisons with the usual instructions (1938 ms; SE = 165), *F*(1, 17) = 77.50, *p* < 0.001, $\eta_{p}^{2}$ = 0.820, MSE = 10622186. As expected, the RTs for the stimulus pairs differed reliably, *F*(3.571, 60.710) = 8.64, *p* < 0.001, $\eta_{p}^{2}$ = 0.337, MSE = 2244378, varying systematically with the ratio of the extents. For the three short line-length pairs with ratios of 1.1, 1.05, and 1.02, overall mean RTs are 2270 (SE = 173), 2395 (SE = 189), and 2596 ms (SE = 208), respectively. For the long pairs with ratios of 1.05, 1.02, and 1.008, overall mean RTs are 2373 (SE = 188), 2680 (SE = 214), and 2696 ms (SE = 250), respectively. The interaction between instructional condition and stimulus pair was also reliable, *F*(4.833, 82.169) = 3.57, *p* < 0.006, $\eta_{p}^{2}$ = 0.174, MSE = 1617378, reflecting the fact that the effect of the ratio-defined discriminative difficulty of the stimulus pairs was generally enhanced for the category-contingent comparisons. Namely, mean RTs are 1869 (SE = 157), 1847 (SE = 157), 2002 (SE = 192), 1929 (SE = 216), 1802 (SE = 140), and 2178 ms (SE = 204) in the usual instructions condition and 2670 (SE = 213), 2942 (SE = 243), 3191 (SE = 257), 3213 ms (SE = 318), 2943 (SE = 243), and 3430 ms (SE = 254) in the category-contingent instructions condition for the 10–11, 20–21, 50–51, 147–150, 200–210, and 250–252 pixel pairs, respectively.

The plots in **Figures 2A,B** are clear in showing that the SCE is very substantially enhanced with the category-contingent comparisons. As such, precisely as in Experiment 1, the three-way interaction involving stimulus pair, instruction direction, and instructional condition is reliable, *F*(3.643, 61.934) = 3.79, *p* < 0.010, $\eta_{p}^{2}$ = 0.182, MSE = 1401542. Indeed, although the two-way interaction of the stimulus pairs and the instruction direction is not actually statistically reliable for the usual instructions, *F*(3.674, 62.457) = 0.75, *p* < 0.554, $\eta_{p}^{2}$ = 0.042, MSE = 1563664, it is for the category-contingent instructions, *F*(5.000, 85.000) = 11.59, *p* < 0.001, $\eta_{p}^{2}$ = 0.405, MSE = 1077759. Regression analyses with the SCE index as dependent variable and the maximal value of the stimulus in each pair as the independent variable show (i.e., the lines in **Figure 2B**) that the slope of the best fitting regression line is steeper with the category-contingent instructions than with the usual instructions, indicating enhanced SCE effects with the category-contingent instructions.

Finally, mean RT reliably decreases across blocks, *F*(3.425, 58.225) = 33.08, *p* < 0.001, $\eta_{p}^{2}$ = 0.661, MSE = 5418036, with mean RTs of 3430 (SE = 201), 2783 (SE = 229), 2425 (SE = 215), 2250 (SE = 210), 2157 (SE = 225), and 1961 ms (SE = 206) for Blocks 1–6, respectively. However, neither the two-way interaction of stimulus pairs with instruction direction nor the three-way interaction involving stimulus pair, instruction direction, and instructional condition interacts reliably with blocks [*F*(16.048, 272.823) = 0.40, *p* < 0.981, $\eta_{p}^{2}$ = 0.023, MSE = 1545599, and *F*(15.196, 258.339) = 0.87, *p* < 0.600, $\eta_{p}^{2}$ = 0.049, MSE = 1731428, respectively]. Hence, neither the overall size of the SCE nor the difference in the sizes of this effect across instructional conditions decreases as mean RT decreases across blocks. On the other hand, the overall difference in mean RT between the category-contingent and usual instructions conditions does decrease reliably across blocks, *F*(4.579, 77.846) = 6.01, *p* < 0.001, $\eta_{p}^{2}$ = 0.261, MSE = 1402815, with differences of 1650, 1166, 969, 1010, 1015, and 952 ms for Blocks 1–6, respectively.

## DISCUSSION

The finding of clear and very substantial increases in the magnitude of the SCE with category-contingent instructions in this study converges nicely with the finding of enhanced SCEs with the instructional format manipulations used by both Shaki et al. (2006) and Petrusic et al. (2008) and is entirely in accord with the evidence-accrual-based theoretical positions developed in either Petrusic (1992) or Leth-Steensen and Marley (2000). Importantly, with respect to the former view, some evidence is available in these results to indicate that decisional processing itself is indeed slowed by the requirement to perform category-contingent comparisons. Namely, that other effects that are typically associated with such processing, such as the stimulus discrimination difficulty effects in Experiment 2, are also enhanced in the category-contingent instructions conditions. Such findings clearly indicate that something more is happening to the processing that is occurring in the category-contingent instruction condition than the simple insertion of an initial stimulus categorization process.

Moreover, with respect to the Leth-Steensen and Marley (2000) model, the semantic facilitation and interference of the instructional pathways that underlies the SCE is assumed to arise because the evidence that is also being accumulated about the endpoint status of the items being compared (see Leth-Steensen and Marley, 2000, for details) also serves to convey categorical-like information about their sizes. This categorical information is then assumed to either semantically facilitate the processing in the congruent instructional pathway or semantically interfere with the processing in the incongruent instructional pathway. Hence, on the basis of this view, a manipulation such as that employed here in the category-contingent instructional condition which explicitly serves to draw attention to the categorical size of the items in a pair during the comparison process would almost certainly be expected to enhance the degree of both semantic facilitation and semantic interference prescribed by the Leth-Steensen and Marley (2000) model, thus greatly enhancing the SCE (a phenomenon which is especially evident for the line-length comparisons in the current Experiment 2 given that a reliable SCE was only obtained for the category-contingent instructions).

With respect to the decreases in RT that occur across blocks of trials, although the effect of instructional condition also decreases across blocks (but mostly across the first three blocks in both experiments), the sizes of the key effects of interest here (i.e., the SCE and the difference in this effect between instructional conditions) essentially did not change. Such results suggest that any enhancements in processing speed with increased practice on the task were not occurring within the evidence accrual process itself but were likely due to speed-ups in processes extraneous to the decisional processing (i.e., perceptual encoding of the instructions and motor response processes as well as any control processes associated with dealing with the initially quite novel category-contingent instructions condition).

It could be argued that the present findings are not at all consistent with what would be expected on the basis of any of the three single-sample accounts for the SCE that were discussed in Petrusic et al. (2008), namely, the expectancy, reference point, and semantic coding views. With respect to the expectancy view (Marschark and Paivio, 1981), the SCE is assumed to arise because the direction specified by the comparative instruction serves to direct the memory search for the relevant features of the items in comparison pair. Within the present paradigm, however, because such a memory search is always explicitly requested in the category-contingent instructional condition before determining the direction of the comparison, no such expectancy effects would be expected to arise and the SCE should actually have been observed to diminish greatly in magnitude. With respect to the reference point view (Jamieson and Petrusic, 1975; Holyoak, 1978), the SCE is assumed to arise because it is easier to compare stimuli that are closer to the reference point than farther from it and also that the reference point used is the one specified by the comparative instruction. Within the present paradigm, however, it is hard to imagine why it wouldn’t be the initial categorization of the items that would then determine the reference points to be used in the subsequent comparison process. Because these reference points would then be the same for both semantically congruent and incongruent stimulus pairs in the category-contingent instructional condition, again, no SCE would be expected in that condition (or at least certainly not an exaggerated one).

Finally, the semantic coding view (Banks, 1977) assumes that the comparison process is made up of a number of additive, serial stages. Namely, that the direction of the instruction is first coded semantically (i.e., S+ or L+), that semantic size codes are then generated for each of the stimulus items (e.g., S and S+ for two small stimuli), that these stimulus codes are then compared to the instructional code, that the stimulus codes are then translated if they are semantically incongruent with the instruction code (e.g., to L+ and L, respectively), and finally that a response is made according to which item’s semantic code matches the instructional code. The SCE is assumed to arise in this model on the basis of whether or not the code translation stage is needed.

With respect to the semantic coding view, as discussed in Petrusic et al. (2008), it could perhaps account for the present findings with the *post hoc* assumption that the increased memory demands in the category-contingent instruction condition slow the code matching and translation processes. However, it occurred to us that the present paradigm actually serves to explicitly invoke most of the processing stages specified by the semantic coding model (in fact, it was probably this model that “primed” us to run this particular study in the first place). Namely, the process of having to first categorize the stimuli in the category-contingent instruction condition is essentially analogous to the semantic code generation stage. Next, asking participants to perform congruent category-contingent decisions (i.e., “small-smaller, but large-larger”) could essentially be regarded as analogous to instructing them to simply identify the more extreme of the two semantic codes and use that information to respond directly. Similarly, asking participants to perform incongruent category-contingent decisions (i.e., “small-larger, but large-smaller”) could essentially be regarded as analogous to instructing them to immediately translate the codes before identifying the more extreme code. Hence, given the inherent similarity between the nature of the processing in the categorical-contingent instructional conditions and that which would normally be expected in the usual instructional conditions according to the semantic coding view, it would not seem to predict that there be a difference in the SCE between these conditions (indeed, it is not even clear according to such a view why there should be any overall differences in RTs between these two conditions).

## CONCLUSION

The current work adds to that reported previously by these same authors and serves to provide further support for an evidence-accrual-based view of the SCE. Indeed, it seems quite clear from all of this work that enhancing the difficulty of determining the direction of the instruction while performing binary comparative judgments effectively serves to slow down the decision process itself, resulting in increases in the SCE.

## Conflict of Interest Statement

The authors declare that the research was conducted in the absence of any commercial or financial relationships that could be construed as a potential conflict of interest.
